# 6-Bromo-1-methyl-1*H*-2,1-benzothia­zin-4(3*H*)-one 2,2-dioxide

**DOI:** 10.1107/S1600536809015980

**Published:** 2009-04-30

**Authors:** Muhammad Shafiq, M. Nawaz Tahir, Islam Ullah Khan, Muhammad Nadeem Arshad, Muhammad Nadeem Asghar

**Affiliations:** a Government College University, Department of Chemistry, Lahore, Pakistan; b University of Sargodha, Department of Physics, Sargodha, Pakistan

## Abstract

In the crystal structure of the title compound, C_9_H_8_BrNO_3_S, the thia­zine ring is in the twisted form. In the crystal, pairs of inter­molecular C—H⋯O hydrogen bonds form inversion dimers with an *R*_2_^2^(8) ring motif. Weak inter­molecular C—H⋯Br and C—H⋯π inter­actions are also present.

## Related literature

For the structures of benzothia­zine derivatives, see: Arshad *et al.* (2008 ▶); Shafiq *et al.* (2008*a* ▶,*b* ▶); Tahir *et al.* (2008 ▶). For the related structure, 6-bromo-1-methyl-1*H*-benzo[*c*][1,2]thia­zin-4(3*H*)-one 2,2-dioxide, see: Shafiq *et al.* (2009 ▶). For hydrogen-bond motifs, see: Bernstein *et al.* (1995 ▶). For puckering parameters, see: Cremer & Pople (1975 ▶). For the synthesis, see: Lombardino (1972 ▶).
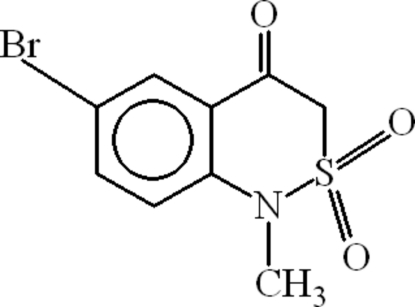


## Experimental

### 

#### Crystal data

C_9_H_8_BrNO_3_S
*M* *_r_* = 290.13
Monoclinic, 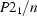

*a* = 5.4577 (3) Å
*b* = 12.6400 (8) Å
*c* = 15.1258 (10) Å
β = 96.204 (2)°
*V* = 1037.35 (11) Å^3^
*Z* = 4
Mo *K*α radiation
μ = 4.15 mm^−1^
*T* = 296 K
0.20 × 0.17 × 0.15 mm

#### Data collection

Bruker Kappa APEXII CCD diffractometer
Absorption correction: multi-scan (*SADABS*; Bruker, 2005 ▶) *T*_min_ = 0.439, *T*_max_ = 0.540
11077 measured reflections
2234 independent reflections
1709 reflections with *I* > 2σ(*I*)
*R*_int_ = 0.032

#### Refinement

*R*[*F* ^2^ > 2σ(*F* ^2^)] = 0.030
*wR*(*F* ^2^) = 0.072
*S* = 1.04
2234 reflections
137 parameters
H-atom parameters constrained
Δρ_max_ = 0.41 e Å^−3^
Δρ_min_ = −0.35 e Å^−3^

### 

Data collection: *APEX2* (Bruker, 2007 ▶); cell refinement: *SAINT* (Bruker, 2007 ▶); data reduction: *SAINT*; program(s) used to solve structure: *SHELXS97* (Sheldrick, 2008 ▶); program(s) used to refine structure: *SHELXL97* (Sheldrick, 2008 ▶); molecular graphics: *ORTEP-3 for Windows* (Farrugia, 1997 ▶) and *PLATON* (Spek, 2009 ▶); software used to prepare material for publication: *WinGX* (Farrugia, 1999 ▶) and *PLATON*.

## Supplementary Material

Crystal structure: contains datablocks global, I. DOI: 10.1107/S1600536809015980/fb2142sup1.cif

Structure factors: contains datablocks I. DOI: 10.1107/S1600536809015980/fb2142Isup2.hkl

Additional supplementary materials:  crystallographic information; 3D view; checkCIF report

## Figures and Tables

**Table 1:** Hydrogen-bond geometry (Å, °)

*D*—H⋯*A*	*D*—H	H⋯*A*	*D*⋯*A*	*D*—H⋯*A*
C3—H3⋯O3^i^	0.93	2.54	3.308 (4)	140
C8—H8*A*⋯O2^ii^	0.97	2.54	3.470 (3)	162
C9—H9*B*⋯O3	0.96	2.41	2.824 (3)	106
C5—H5⋯Br1^iii^	0.93	2.94	3.871 (3)	175
C9—H9*A*⋯Br1^iv^	0.96	3.01	3.871 (2)	150
C9—H9*C*⋯*Cg*1^v^	0.96	2.83	3.449 (3)	123

Symmetry codes: (i) 
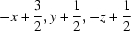
; (ii) 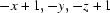; (iii) 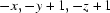; (iv) 
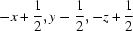
; (v) 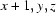. *Cg*1 is the centroid of the C1–C6 ring.

**Fig. 1.**
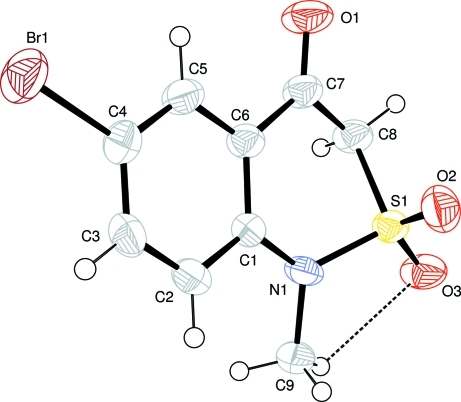
The title compound, with the atom-numbering scheme. The displacement ellipsoids are drawn at the 50% probability level. The H-atoms are shown by small circles of arbitrary radius. The dotted lines show the intramolecular H-bonds.

**Fig. 2.**
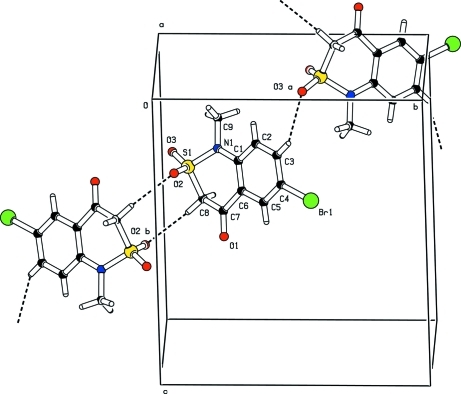
A section of the title structure showing the dimers bind by the hydrogen bonds.

## supplementary crystallographic information

### Comment

We have reported crystal structures of the synthesized derivatives of the benzothiazine molecule (Shafiq *et al.*, 2008*a*; Shafiq *et al.*, 2008*b*; Tahir *et al.*, 2008; Arshad *et al.*, 2008). Here we report the title compound (I), (Fig. 1), that belongs to this series of the structures.

(I) is closely related to the crystal structure of 6-bromo-1-methyl-1*H*-benzo[*c*][1,2]thiazin-4(3*H*)-one 2,2-dioxide, (II), (Shafiq *et al.*, 2009). (I) and (II) differ by the presence of the methyl and ethyl groups at the N-atom, respectively. The bromo-substituted benzene ring A (C1—C6) is planar with Br deviated by 0.064 (4) Å from the mean plane. The thiazine ring B (S1/N1/C1/C6—C8) is in the twisted form, with the maximum puckering amplitude Q_T_ = 0.577 (2) Å (Cremer & Pople, 1975). The title molecules form dimers interconnected by a pair of the intermolecular H-bonds C8–H8A···O2^i^ [symmetry code: i = -*x* + 1, -*y*, -*z* + 1] with the *R*_2_^2^(8) ring motif (Bernstein *et al.*, 1995), (Tab. 1, Fig. 2). The dimers are linked to each other forming helices through the other intermolecular H-bonding C3–H3···O3^ii^ [symmetry code: ii = -*x* + 3/2, *y* + 1/2, -*z* + 1/2]. The molecules are also stabilized due to C—H···π-electron interaction with the benzene group and intermolecular C—H···Br interactions (Tab. 1).

### Experimental

The title compound was prepared in a three step scheme following the reported procedure (Lombardino, 1972). In the first step, methyl-2-amino-5-bromobenzoate (92 mg, 4 mmol) was put in dichloromethane (10 ml) and this mixture was introduced into a round bottom flask. A solution of methanesulfonyl chloride (550 mg, 4.8 mmol) in dichloromethane (10 ml) was slowly added (10-15 minutes) to this mixture. The mixture was stirred at 60–70 °C for 2–3 days keeping pH of the mixture alkaline by triethylamine. After the completion of the reaction, the solvent was evaporated under reduced pressure to get methyl-5-bromo-2-[(methylsulfonyl)amino] benzoate.

In the second step, methyl-5-bromo-2-[(methylsulfonyl)amino] benzoate (1.02 g, 3.3 mmol) was introduced into 5 ml of *N*,*N*-dimethylformamide (DMF). The mixture was added to a suspension of NaH (158.38 mg, 6.6 mmol) in DMF (10 ml). The mixture was stirred at room temperature for 14–16 h. After that, methyl-5-bromo-2-[methyl(methylsulfonyl)amino]benzoate was obtained.

In the third step methyl-5-bromo-2-[methyl(methylsulfonyl)amino]benzoate was cyclized. Therefore methyl-5-bromo-2-[methyl(methylsulfonyl)amino]benzoate (418.83 mg, 1.3 mmol) was introduced in DMF (5 ml) and added to the suspension of NaH (59.99 mg, 2.5 mmol) in DMF (10 ml). The mixture was stirred at room temperature for 3–4 h. Then the reaction mixture was poured into ice and clear solution was obtained. The pH of this solution was adjusted between 5–6. The precipitated crude product was recrystallized from ethanol. Yellow needle-shaped crystals of the title compound of suitable size for structure analysis were grown in this way.

### Refinement

Though all the hydrogens were discernible in the difference electron density map, the H-atoms were situated into idealized positions, with C-H = 0.93, 0.96 and 0.97 Å for aryl, methyl and methylene H, resepctively, and constrained to ride on their parent atoms, with U_iso_(H) = xU_eq_(C), where x = 1.5 for methyl and 1.2 for other carrier atoms.

### Crystal data

**Table:**

C_9_H_8_BrNO_3_S	*F*(000) = 576
*M**_r_* = 290.13	*D*_x_ = 1.858 Mg m^−^^3^
Monoclinic, *P*2_1_/*n*	Mo *K*α radiation, λ = 0.71073 Å
Hall symbol: -P 2yn	Cell parameters from 2234 reflections
*a* = 5.4577 (3) Å	θ = 2.1–27.0°
*b* = 12.6400 (8) Å	µ = 4.15 mm^−^^1^
*c* = 15.1258 (10) Å	*T* = 296 K
β = 96.204 (2)°	Prism, yellow
*V* = 1037.35 (11) Å^3^	0.20 × 0.17 × 0.15 mm
*Z* = 4	

### Data collection

**Table:**

Bruker Kappa APEXII CCD diffractometer	2234 independent reflections
Radiation source: fine-focus sealed tube	1709 reflections with *I* > 2σ(*I*)
graphite	*R*_int_ = 0.032
Detector resolution: 7.40 pixels mm^-1^	θ_max_ = 27.0°, θ_min_ = 2.1°
ω scans	*h* = −6→6
Absorption correction: multi-scan (*SADABS*; Bruker, 2005)	*k* = −16→16
*T*_min_ = 0.439, *T*_max_ = 0.540	*l* = −18→19
11077 measured reflections	

### Refinement

**Table:**

Refinement on *F*^2^	Primary atom site location: structure-invariant direct methods
Least-squares matrix: full	Secondary atom site location: difference Fourier map
*R*[*F*^2^ > 2σ(*F*^2^)] = 0.030	Hydrogen site location: difference Fourier map
*wR*(*F*^2^) = 0.072	H-atom parameters constrained
*S* = 1.04	*w* = 1/[σ^2^(*F*_o_^2^) + (0.0309*P*)^2^ + 0.4349*P*] where *P* = (*F*_o_^2^ + 2*F*_c_^2^)/3
2234 reflections	(Δ/σ)_max_ = 0.001
137 parameters	Δρ_max_ = 0.41 e Å^−^^3^
0 restraints	Δρ_min_ = −0.35 e Å^−^^3^
31 constraints	

### Special details

**Table:**

Geometry. All e.s.d.'s (except the e.s.d. in the dihedral angle between two l.s. planes) are estimated using the full covariance matrix. The cell e.s.d.'s are taken into account individually in the estimation of e.s.d.'s in distances, angles and torsion angles; correlations between e.s.d.'s in cell parameters are only used when they are defined by crystal symmetry. An approximate (isotropic) treatment of cell e.s.d.'s is used for estimating e.s.d.'s involving l.s. planes.
Refinement. Refinement of *F*^2^ against ALL reflections. The weighted *R*-factor *wR* and goodness of fit *S* are based on *F*^2^, conventional *R*-factors *R* are based on *F*, with *F* set to zero for negative *F*^2^. The threshold expression of *F*^2^ > σ(*F*^2^) is used only for calculating *R*-factors(gt) *etc*. and is not relevant to the choice of reflections for refinement. *R*-factors based on *F*^2^ are statistically about twice as large as those based on *F*, and *R*- factors based on ALL data will be even larger.

### Fractional atomic coordinates and isotropic or equivalent isotropic displacement parameters (Å^2^)

**Table:**

	*x*	*y*	*z*	*U*_iso_*/*U*_eq_	
Br1	0.06150 (6)	0.57510 (2)	0.36727 (2)	0.05871 (14)	
S1	0.74473 (12)	0.10243 (5)	0.40740 (4)	0.03706 (17)	
O1	0.3779 (4)	0.23972 (17)	0.56688 (14)	0.0606 (6)	
O2	0.5116 (4)	0.05733 (15)	0.37692 (14)	0.0529 (5)	
O3	0.9592 (4)	0.03989 (16)	0.40469 (14)	0.0555 (6)	
N1	0.7895 (4)	0.21290 (17)	0.35343 (14)	0.0385 (5)	
C1	0.6129 (4)	0.29366 (19)	0.35581 (16)	0.0326 (6)	
C2	0.5631 (5)	0.3622 (2)	0.28382 (19)	0.0403 (6)	
H2	0.6430	0.3525	0.2332	0.048*	
C3	0.3980 (5)	0.4437 (2)	0.2866 (2)	0.0430 (7)	
H3	0.3650	0.4882	0.2378	0.052*	
C4	0.2814 (5)	0.4595 (2)	0.36193 (19)	0.0409 (6)	
C5	0.3200 (5)	0.3919 (2)	0.43312 (19)	0.0416 (6)	
H5	0.2368	0.4024	0.4829	0.050*	
C6	0.4846 (4)	0.3073 (2)	0.43087 (17)	0.0362 (6)	
C7	0.5129 (5)	0.2357 (2)	0.50864 (18)	0.0407 (6)	
C8	0.7204 (5)	0.1553 (2)	0.51326 (17)	0.0419 (6)	
H8A	0.6884	0.0989	0.5540	0.050*	
H8B	0.8745	0.1891	0.5355	0.050*	
C9	0.9601 (4)	0.2116 (2)	0.28524 (19)	0.0415 (6)	
H9A	0.8798	0.1812	0.2316	0.062*	
H9B	1.1024	0.1701	0.3057	0.062*	
H9C	1.0102	0.2826	0.2737	0.062*	

### Atomic displacement parameters (Å^2^)

**Table:**

	*U*^11^	*U*^22^	*U*^33^	*U*^12^	*U*^13^	*U*^23^
Br1	0.0680 (2)	0.0468 (2)	0.0609 (2)	0.02306 (14)	0.00505 (16)	0.00177 (15)
S1	0.0439 (3)	0.0358 (4)	0.0327 (4)	0.0072 (3)	0.0098 (3)	0.0042 (3)
O1	0.0815 (14)	0.0670 (14)	0.0380 (12)	0.0261 (11)	0.0282 (11)	0.0127 (11)
O2	0.0603 (12)	0.0441 (12)	0.0541 (14)	−0.0125 (9)	0.0050 (10)	0.0006 (10)
O3	0.0652 (12)	0.0557 (13)	0.0489 (13)	0.0284 (10)	0.0214 (10)	0.0133 (10)
N1	0.0420 (11)	0.0415 (13)	0.0350 (13)	0.0070 (9)	0.0179 (10)	0.0092 (10)
C1	0.0345 (12)	0.0319 (14)	0.0317 (15)	−0.0013 (10)	0.0052 (10)	0.0010 (11)
C2	0.0446 (14)	0.0398 (15)	0.0382 (17)	0.0001 (11)	0.0127 (12)	0.0070 (12)
C3	0.0523 (15)	0.0346 (15)	0.0423 (17)	−0.0010 (12)	0.0061 (13)	0.0102 (12)
C4	0.0426 (13)	0.0343 (14)	0.0453 (18)	0.0060 (11)	0.0026 (12)	−0.0001 (13)
C5	0.0483 (15)	0.0431 (15)	0.0347 (16)	0.0084 (12)	0.0104 (12)	−0.0017 (13)
C6	0.0415 (13)	0.0386 (15)	0.0288 (15)	0.0030 (11)	0.0051 (11)	0.0017 (11)
C7	0.0502 (14)	0.0431 (16)	0.0296 (15)	0.0072 (12)	0.0080 (12)	0.0006 (12)
C8	0.0523 (15)	0.0464 (17)	0.0274 (15)	0.0104 (12)	0.0058 (12)	0.0057 (12)
C9	0.0403 (13)	0.0444 (16)	0.0425 (17)	−0.0026 (11)	0.0171 (12)	−0.0018 (13)

### Geometric parameters (Å, °)

**Table:**

Br1—C4	1.898 (3)	C3—C4	1.379 (4)
S1—O3	1.4169 (19)	C3—H3	0.9300
S1—O2	1.424 (2)	C4—C5	1.372 (4)
S1—N1	1.649 (2)	C5—C6	1.400 (3)
S1—C8	1.753 (3)	C5—H5	0.9300
O1—C7	1.209 (3)	C6—C7	1.479 (4)
N1—C1	1.407 (3)	C7—C8	1.518 (3)
N1—C9	1.463 (3)	C8—H8A	0.9700
C1—C2	1.395 (3)	C8—H8B	0.9700
C1—C6	1.407 (3)	C9—H9A	0.9600
C2—C3	1.373 (4)	C9—H9B	0.9600
C2—H2	0.9300	C9—H9C	0.9600
			
O3—S1—O2	118.63 (13)	C4—C5—C6	120.1 (2)
O3—S1—N1	106.93 (11)	C4—C5—H5	120.0
O2—S1—N1	110.70 (12)	C6—C5—H5	120.0
O3—S1—C8	112.54 (13)	C5—C6—C1	119.3 (2)
O2—S1—C8	107.17 (13)	C5—C6—C7	117.4 (2)
N1—S1—C8	99.15 (12)	C1—C6—C7	123.2 (2)
C1—N1—C9	121.1 (2)	O1—C7—C6	122.3 (2)
C1—N1—S1	117.60 (16)	O1—C7—C8	120.3 (2)
C9—N1—S1	118.62 (17)	C6—C7—C8	117.4 (2)
C2—C1—N1	120.5 (2)	C7—C8—S1	110.06 (18)
C2—C1—C6	118.8 (2)	C7—C8—H8A	109.6
N1—C1—C6	120.8 (2)	S1—C8—H8A	109.6
C3—C2—C1	121.1 (2)	C7—C8—H8B	109.6
C3—C2—H2	119.5	S1—C8—H8B	109.6
C1—C2—H2	119.5	H8A—C8—H8B	108.2
C2—C3—C4	119.8 (3)	N1—C9—H9A	109.5
C2—C3—H3	120.1	N1—C9—H9B	109.5
C4—C3—H3	120.1	H9A—C9—H9B	109.5
C5—C4—C3	120.9 (2)	N1—C9—H9C	109.5
C5—C4—Br1	119.3 (2)	H9A—C9—H9C	109.5
C3—C4—Br1	119.8 (2)	H9B—C9—H9C	109.5
			
O3—S1—N1—C1	−172.74 (19)	Br1—C4—C5—C6	−179.0 (2)
O2—S1—N1—C1	56.7 (2)	C4—C5—C6—C1	1.2 (4)
C8—S1—N1—C1	−55.7 (2)	C4—C5—C6—C7	−178.3 (2)
O3—S1—N1—C9	25.6 (2)	C2—C1—C6—C5	−3.1 (4)
O2—S1—N1—C9	−105.0 (2)	N1—C1—C6—C5	176.5 (2)
C8—S1—N1—C9	142.7 (2)	C2—C1—C6—C7	176.5 (2)
C9—N1—C1—C2	12.8 (4)	N1—C1—C6—C7	−3.9 (4)
S1—N1—C1—C2	−148.4 (2)	C5—C6—C7—O1	9.9 (4)
C9—N1—C1—C6	−166.8 (2)	C1—C6—C7—O1	−169.7 (3)
S1—N1—C1—C6	32.0 (3)	C5—C6—C7—C8	−170.1 (2)
N1—C1—C2—C3	−177.5 (2)	C1—C6—C7—C8	10.3 (4)
C6—C1—C2—C3	2.0 (4)	O1—C7—C8—S1	139.9 (2)
C1—C2—C3—C4	0.9 (4)	C6—C7—C8—S1	−40.0 (3)
C2—C3—C4—C5	−2.8 (4)	O3—S1—C8—C7	170.50 (19)
C2—C3—C4—Br1	177.9 (2)	O2—S1—C8—C7	−57.3 (2)
C3—C4—C5—C6	1.7 (4)	N1—S1—C8—C7	57.8 (2)

### Hydrogen-bond geometry (Å, °)

**Table:**

*D*—H···*A*	*D*—H	H···*A*	*D*···*A*	*D*—H···*A*
C3—H3···O3^i^	0.93	2.54	3.308 (4)	140
C8—H8A···O2^ii^	0.97	2.54	3.470 (3)	162
C9—H9B···O3	0.96	2.41	2.824 (3)	106
C5—H5···Br1^iii^	0.93	2.94	3.871 (3)	175
C9—H9A···Br1^iv^	0.96	3.01	3.871 (2)	150
C9—H9C···Cg1^v^	0.96	2.83	3.449 (3)	123

Symmetry codes: (i) −*x*+3/2, *y*+1/2, −*z*+1/2; (ii) −*x*+1, −*y*, −*z*+1; (iii) −*x*, −*y*+1, −*z*+1; (iv) −*x*+1/2, *y*−1/2, −*z*+1/2; (v) *x*+1, *y*, *z*.
